# Separate segments within the deltoid muscle: Anatomical variants or wishful thinking?

**DOI:** 10.4103/0973-6042.42583

**Published:** 2008

**Authors:** E. Audenaert, E. Barbaix

**Affiliations:** Department of Orthopedic Surgery and Traumatology, Ghent University Hospital, De Pintelaan 185, 9000 Ghent, Belgium; 1 Department of Human Anatomy, Ghent University Hospital, De Pintelaan 185, 9000 Ghent, Belgium

Amongst the numerous publications on anatomic variations of the deltoid that were reviewed by Bergman *et al.*, several concern the absence of one or more bundles of the muscle (most frequently the clavicular bundle or part of the acromial bundles) or the existence of supernumerary posterior bundles.[1]

Milianitch and Spiridonovitch, in their study of 200 Serbian bodies, found a clear division of the deltoid muscle in 30 specimens; in 26 cases the spinal part was separated.[2] They also found three posterior supernumerary fascicles. According to Mori the subdivision of the muscle is much more frequent among the Japanese (with over 25% of cases showing complete separations).[1]

**Figure 1:**
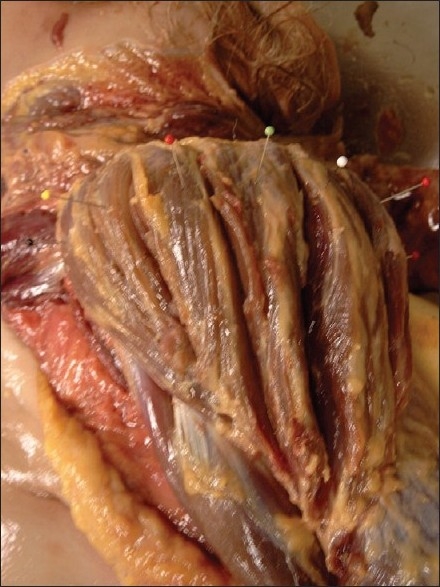
Dissection of the deltoid muscle in partial and fully separated segments usually results in about seven segments as originally described by Albinus

It appears that the deltoid muscle usually develops in distinctly separated but interconnected segments. Albinus probably was the first anatomist to describe the deltoid muscle in seven interconnected but easily separated (‘*ex septem portionibus, inter se conjunctis, sed non difficulter distinguendis*’) segments.[3] In 1908, Frohse and Fränkel also described seven portions and this description was also adopted by Fick in his extensive work on biomechanics.[4–5] The dissection of these bundles is to a large extent a matter of interpretation and overenthusiastic dissection could well create additional or fully separated bundles [Figure 1]. Are these bundles real or do they reflect wishful thinking? According to Testut (1884), there are several arguments in support of their being real. In several animal species, the deltoid muscle is normally subdivided into separate bundles.[6] Septation and distinct fascicles (e.g., coxofemoral muscle) are frequent in the gluteus maximus muscle which, from a developmental point of view, is similar to the deltoid muscle. In muscular and in obese bodies, septae are easily visible, whereas in older specimens with hypotrophic muscles it is more difficult to make out. In body builders, bulging parts of the muscle can be seen separated by sulci.

In addition to the existing anatomical data on the segmented nature of the deltoid muscle, recent mechanomyographic studies have revealed that the deltoid muscle is composed of at least seven functional muscle segments, all of which have the potential to be - at an important level - independently coordinated by the central nervous system. This phenomenon, which has been previously termed ‘functional differentiation,’ explains how single muscles may produce a variety of force vectors through selective activation / deactivation of motor units within its constituent segments.[7] This allows the central nervous system to fine tune the activity of the deltoid muscle motor units. It has also been shown that there is a significant intersegment variation in fiber-type composition, contractile properties, and architectural characteristics, all of which result in important differences in the biomechanical properties of these different functional segments.[8]

The case study presented in this issue of the journal of an anatomical variant of the deltoid muscle confirms, from an anatomical point of view, the above findings of functional and anatomical segmentation of the deltoid muscle.[9] Whether the case represents a rare finding or not, is a question that remains open for further discussion
